# Review article: Primer for clinical researchers on innovative trial designs for emergency medicine

**DOI:** 10.1111/1742-6723.14532

**Published:** 2024-11-18

**Authors:** Katherine J Lee, Melissa Middleton, Robert K Mahar

**Affiliations:** ^1^ Clinical Epidemiology and Biostatistics Unit Murdoch Children's Research Institute Melbourne Victoria Australia; ^2^ Department of Paediatrics University of Melbourne Melbourne Victoria Australia; ^3^ Biostatistics Unit, Centre for Epidemiology and Biostatistics, Melbourne School of Population and Global Health, Faculty of Medicine, Dentistry, and Health Sciences University of Melbourne Melbourne Victoria Australia; ^4^ Methods and Implementation Support for Clinical and Health Sciences Research Hub University of Melbourne Melbourne Victoria Australia

**Keywords:** adaptive trial, cluster randomised, platform trial, randomised trial

## Abstract

Randomised trials have long been recognised as the gold standard research tool for evidence‐based medicine. The past decade has seen the emergence of several innovative trial designs that are revolutionising how trials are conducted. These innovative designs enable more efficient, pragmatic trials that can address complex research questions which were previously not possible. In this paper, we provide an overview of the key innovative designs that are likely to be useful in the emergency medicine context, namely cluster crossover and stepped wedge designs, sequential multiple assignment randomised trial (SMART) designs, and platform trials. We describe the main features of each design, outline their pros and cons, and describe when they may or may not be useful. We also provide examples of these innovative designs in contexts that are relevant to emergency medicine.

## Graphical abstract

Randomised trials are the cornerstone of evidence‐based medicine, but traditional designs can be inefficient and have limited scope. This paper provides an overview of innovative clustered, sequential and platform designs which enable more efficient, pragmatic trials that can address complex research questions.

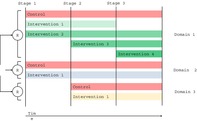

**Key findings**

- Randomised trials are the cornerstone of evidence‐based medicine.
- Innovative designs enable more efficient, pragmatic trials that can address complex research questions.
- It is important that researchers are aware of these novel designs so they can choose the most appropriate design to answer important research questions as effectively and efficiently as possible.

## Introduction

Randomised trials have long been recognised as the gold standard research tool for evidence‐based medicine.^1^ The standard, parallel group, design randomises participants between two or more groups, often an *intervention* and a *control* group, the latter being either a placebo, standard of care or no intervention. The aim of such a study is to determine the effectiveness of the intervention compared to the control. With standard parallel group designs, the sample size is determined *a priori* based on the research question of interest (whether testing superiority, equivalence or non‐inferiority of the intervention), assumptions about the distribution of the outcome in the control group, the minimal clinically significant difference, and the desired type I error (false positive rate) and power, and the trial continues until the sample size is reached at which time the trial data are analysed and reported.

Despite the familiarity of parallel group designs, they can be inefficient and are restricted to fairly simple settings. Many innovative trial designs have emerged over the past two decades that are revolutionising how trials are conducted. These innovative designs enable more efficient, pragmatic trials that can address complex research questions which are not possible using standard parallel group designs and are responsive to changing landscape of evidence regarding potential interventions. The aim of this paper is to provide an overview of the key innovative designs likely to be useful in emergency medicine, namely cluster crossover and stepped wedge designs, sequential multiple assignment randomised trials (SMART), and platform trials. We describe the main features of each design, outline the pros and cons, and describe when they may be useful. An overview of these designs is provided in Table 1.

**TABLE 1:** Summary of novel trial designs

Design	Description	Pros	Cons	When to use
Cluster crossover	Cluster randomised design in which clusters switch back and forth between the interventions	Typically, more efficient and requires fewer clusters than standard cluster randomised trials because comparisons are made within clusters Can estimate cluster‐specific effects	There may be a carryover effect between periods Need to allow for within‐cluster within period and within‐cluster between period correlation in the sample size calculation and the statistical analysis	When an intervention is delivered at the cluster level and can be easily implemented and removed When there is unlikely to be a carryover effect of the intervention(s) When there are a finite number of clusters and want to increase statistical power
Stepped wedge	Cluster randomised design where an intervention is at the cluster level and the intervention is rolled out in stages across the clusters	Attractive to policy makers and service providers because all clusters receive the intervention Typically, more efficient and requires fewer clusters than standard cluster randomised trials because comparisons are made within clusters Can estimate cluster‐specific effects Allows investigators to examine how the impact of the intervention develops over time once it has been introduced into a cluster	The effect of the intervention may be confounded with calendar time Sample size calculations and analysis must allow for both the clustering of observations and the confounding effect of time Often open‐labelled Requires cooperation and commitment from the clusters to be ready to initiate the intervention & remain in the trial until its conclusion	When randomisation to the control or intervention arm is not possible but randomisation to a date of initiation is When an intervention occurs at the cluster level, all clusters want or are planned to receive the intervention, and once the intervention has been initiated it cannot be removed When an intervention is being rolled out across clusters anyway When there is access to statistical expertise to allow for the clustering and temporal trends
Sequential multiple assignment randomised trial	Trial where participants are randomised at multiple time‐points to a set of interventions conditional on the participant's intermediate outcome	Patient often rerandomized if treatment fails (increases patient acceptability) Can allow optimisation of dynamic treatment regimens or stage‐specific decision rules Can capture synergistic effects of sequential treatments	Unfamiliar Statistically complicated Often require substantial sample sizes to identify optimal dynamic treatment regimens Limited to small number of stages and/or treatments	When interested in dynamic treatment regimens When there is appropriate statistical expertise to design and analyses the trial
Platform	Examine the efficacy of multiple interventions simultaneously, across multiple domains (treatment modalities) and possibly within different subgroups of participants, with rules in place to adaptively add or remove arms based on interim efficacy data, under a single “master” protocol	Can answer multiple research questions simultaneously Greater statistical efficiency than conventional designs May allow fewer patients to be exposed to potentially inferior treatments May enable improved understanding of drug effects, e.g. in subgroups Greater acceptability to stakeholders due to added flexibility Economies of scale: reduces duplication and variable cost of adding research questions	Substantial investment at outset Documentation takes much longer to write than for a conventional trial Heavily reliant on ongoing specialist expertise, including biostatistics Analysis is much more complex – need to account for design features Typically, only useful if the outcome is short term, relative to recruitment Sample size requires simulation, is often not fixed *a priori* and is typically perpetual Faces logistical challenges particularly around data management	If the outcome of interest is relatively short term relative to the recruitment period When there are multiple therapies available When there are likely to be new therapies becoming available If there is the potential for heterogeneity of treatment response across subgroups When the team has access to expertise (e.g. statistical) and resources to design and implement these trial designs

## Cluster randomised designs

When interventions are implemented or delivered at a group level (e.g. within a hospital), or there may be contamination between participants randomised to different interventions (due to sharing of information or practice across participants), it may be preferable to use a cluster randomised design.

In a cluster randomised trial, randomisation occurs at the group‐ or cluster‐level, with each cluster randomised to the intervention or control group.^2^, ^3^, ^4^ Observations within a cluster are expected to be correlated since participants within a cluster are likely to be more similar than participants in different clusters. This correlation means standard statistical approaches are not valid as they underestimate the uncertainty in the estimated treatment effect. Therefore, clustering must be accounted for in the sample size calculation, as ignoring the clustering will result in an underpowered trial,^5^, ^6^ and in the statistical analysis.^7^ Allowing for clustering in the sample size calculation involves estimating the within‐cluster correlation which can be difficult without existing data.

The use of cluster randomised trials can be limited by the number of clusters available, as few clusters can limit statistical power, and if clusters do not want to be randomised to the control arm. Recent years have seen the emergence of cluster crossover and stepped wedge designs which overcome these limitations.

Case study: A trial looking at the effectiveness of targeted interventions compared with passive dissemination of an evidence‐based bronchiolitis guideline where the interventions were delivered at the hospital level.^8^

### Cluster crossover trials

**Figure 1:**
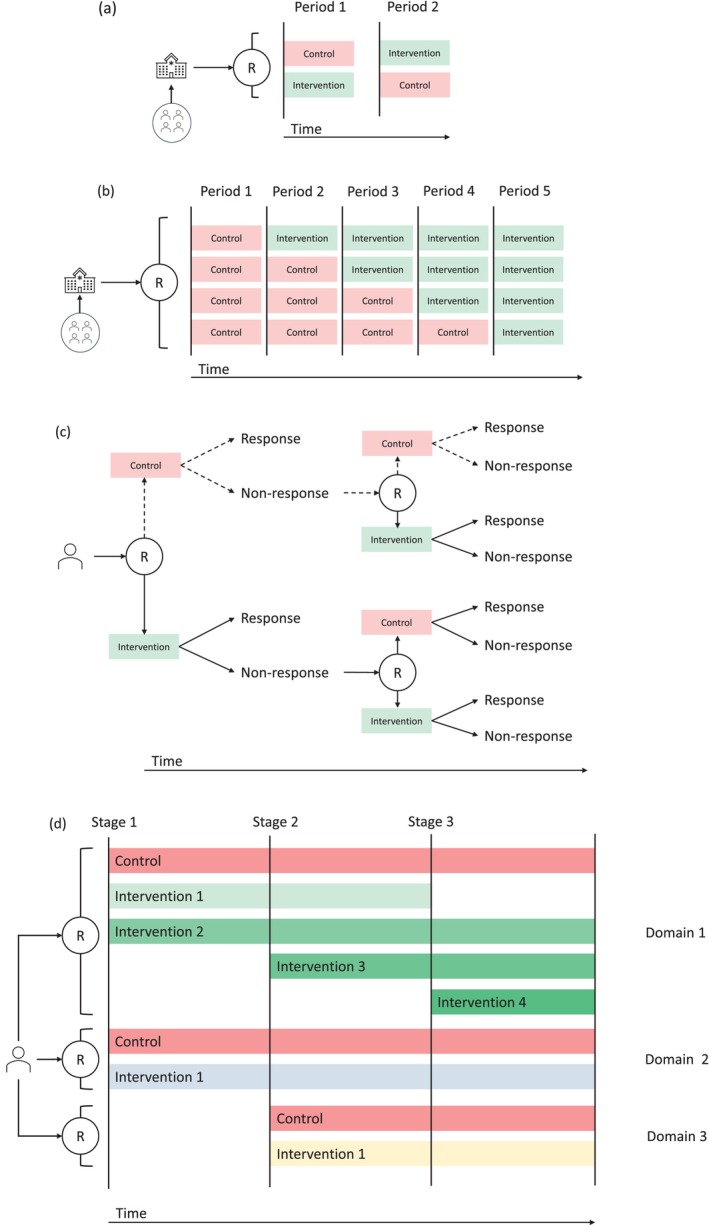
Schematic of innovative trial designs. (a) Cluster crossover trial with two intervention arms and two periods. (b) Stepped wedge trial with four clusters and five periods. (c) Sequential multiple assignment randomised trial with two stages, where non‐responders are re‐randomised to intervention or control. (d) Platform trial with three domains*. R, randomisation. *The platform starts with two domains. Domain 1 initially randomises participants between three arms. A new intervention (Intervention 3) is added to Domain 1 in Stage 2, and one intervention is stopped (Intervention 1) and another new intervention (Intervention 4) is added in Stage 3. Domain 2 randomises participants between two arms throughout the trial. A new domain (Domain 3) is added in Stage 2 and randomises participants between two arms until the end of the trial.

In cluster crossover trials, randomisation occurs at the cluster level, but rather than clusters being randomised between interventions, all clusters receive all interventions but are randomised to the order in which the interventions are received.^9^ For example, in a two‐arm, two‐period cluster crossover trial of an intervention *versus* control, half of the clusters would be randomised to receive the intervention in a first period of time and the control in a second period, and the other half to receive the control in the first period and the intervention in the second (Fig. 1a). This design can be extended with additional interventions and/or time periods. In a cluster crossover trial, the outcome can be a single measurement from each participant, with different participants in each period, from repeat measurements on the same cohort of individuals in each period, or a mixture of the two.^10^

The major advantage of cluster crossover designs is that the intervention group comparisons can be made within clusters. The within‐cluster comparison controls for cluster‐level characteristics and can remove some of the uncertainty in the estimation of the intervention effect reducing the required sample size.^9^ It also means the effectiveness of the intervention can be explored within clusters. One disadvantage of cluster crossover designs is that there may be a carryover effect between periods (which occurs if the intervention has a lasting effect). If one or more interventions are expected to have a carryover effect, it may be necessary to include a washout period before starting the second intervention. Either way, the potential for a period effect must be allowed for in the statistical analysis. A second disadvantage is that the sample size calculation for a cluster crossover study requires estimation of the correlation among observations within a cluster in the same period and among observations within a cluster in different periods. Finally, crossover designs are restricted to examining interventions that can be easily implemented and removed.

Case study: The Sodium Chloride or Plasmalyte‐148 Evaluation in Severe Diabetic Ketoacidosis (SCOPE‐DKA) trial randomised clusters to give plasmalyte‐148 or normal saline as fluid therapy for patients admitted to the intensive care unit with severe Diabetic Ketoacidosis.^11^ Clusters used one intervention for a 6‐month period before crossing over to the alternative treatment, with a 1‐month washout between periods.

### Stepped wedge trials

In stepped wedge trials, all clusters initially receive the control and the intervention is rolled out at regular intervals (periods) until all clusters have received the intervention.^12^, ^13^ The order in which the intervention is initiated across clusters is randomised so the only difference between clusters is their ordering (reducing potential confounding). The trial ends with a period when all clusters receive the intervention (Fig. 1b). As with cluster crossover trials, the outcome in a stepped wedge trial can be a single measurement from each participant, with different participants in each stage, from repeat measurements on the same cohort of participants in each period, or a mixture of the two.^13^ When using a stepped wedge design, ideally all clusters would be identified at the beginning of the study and would not know when they are due to initiate the intervention. In practice, clusters may be recruited throughout the study for logistical reasons and may want to know when they are initiating the intervention so they have time to prepare.

Like cluster crossover trials, stepped wedge designs are more efficient than conventional cluster randomised trials because intervention comparisons are made within clusters, and they enable the investigation of cluster‐specific effects. Another advantage of stepped wedge designs is they can be used to investigate how the intervention effect changes over time.^13^ The disadvantages of stepped wedge designs are there may be temporal trends in outcomes which can have a confounding effect as the control occurs before the intervention in all clusters. The effect of time must therefore be considered in the sample size calculation and the analysis,^10^ and can make interpretation difficult if there are strong temporal trends. Like cluster crossover designs the within‐cluster within‐period and within‐cluster between‐period correlations must be incorporated into the sample size calculation and statistical analysis.^13^

Stepped wedge trials can be used when all clusters want to, or are planned to, receive the intervention, and where, once implemented, it will not be removed. It is a pragmatic study design that is often used to evaluate the effectiveness of an intervention that is being implemented anyway. Such designs are often used by policy makers and service managers to evaluate service delivery interventions, where the decision makers have the belief that the intervention is effective. In some cases, stepped wedge designs can be used to overcome logistical constraints, for example where the intervention is time/resource intensive to implement and must be implemented in stages.

Case study: The Enhanced Peri‐Operative Care for High‐risk patients (EPOCH) trial considered the implementation of a national quality improvement programme to promote a perioperative care pathway for patients undergoing emergency abdominal surgery. Fifteen geographical clusters were identified and the trial broken into 17 five‐week periods, with the intervention rolled out to one cluster every period.^14^

## Sequentially multiple assignment randomised trials

Patients with chronic or progressive conditions typically have needs that evolve over time, often in response to evolving patient histories and responses to previous treatments. A dynamic treatment regimen (DTR) is a formalisation of a multi‐stage, branching treatment pathway.^15^ For example, a simple DTR might recommend starting treatment with most common standard of care and if it works to continue treatment, but if it fails switch to something else. Although the standalone efficacy of each treatment in the sequence might be well‐established, the efficacy of the DTR may not.

Sequential multiple assignment randomised trials (SMART) provide a method for evaluating the efficacy of DTRs. A SMART randomises participants to an initial treatment and, depending on their response, re‐randomises them to a subsequent treatment^16^ (Fig. 1c). A SMART will, by definition, have multiple embedded DTRs, with the aim of identifying which is the optimal DTR and/or the optimal stage‐specific treatment. Note SMARTs are different to designs that simply randomise participants to different sequences of treatments which would provide a comparison of treatment sequences, not DTRs.

The advantage of SMARTs is they can be used to compare the effectiveness of complex treatment pathways, which can include multiple sequential treatments with synergistic effects and the tailoring of treatments depending on intermediate outcomes. SMARTs, however, face technical challenges. Sample size calculations and analysis methods are often unfamiliar and complicated, as they must account for participants contributing information to multiple DTRs when comparing DTRs or for patient histories and expected outcomes at subsequent stages when evaluating stage‐specific effects. Furthermore, large sample sizes may be needed to compare DTRs, particularly if the SMART involves more than two stages or many treatments. A common workaround is to power the study for the first‐stage treatment comparison as the primary aim, and to evaluate the DTRs as secondary aim. Although underpowered, this may provide preliminary evidence for a head‐to‐head trial of candidate DTRs, which would be amenable to a parallel‐group design.

Case study: A trial in the management of alcohol use and violent behaviours in adolescents and emerging adults presenting to the ED in which participants are first randomised between receiving text messages or a health coach as a behavioural intervention. Responders are then re‐randomised between maintenance or a *step down* of the initial intervention, and non‐responders re‐randomised between either maintenance or a *step up* of the initial intervention.^17^

## Adaptive designs

Standard parallel group designs have a fixed sample size and analyse the trial data once the sample size is reached. If recruitment is slow, this can mean an unacceptable amount of time to reach a conclusion. Furthermore, if the population is small, the required sample size may not be feasible. Even if a trial is feasible and quick to recruit, if an early look at the efficacy data allows a definitive conclusion, it can avoid randomising more participants than necessary to an inferior treatment and may have globally significant impact.

Adaptive designs overcome many of the deficiencies of fixed designs by allowing the trial to be modified based on accumulating trial data according to one or more of the following design components^18^, ^19^:

- Early stopping: The trial (or an arm of a multi‐arm trial) can be stopped early where there is a strong signal of efficacy (or futility, that is unlikely to reach any conclusion).
- Response adaptive randomisation: A randomisation procedure that reweights the trials randomisation allocation ratios in favour of better performing interventions based on accumulated trial data.
- Sample size re‐estimation: The required sample size can be re‐estimated based on revised assumptions about the average outcome in the control group and/or the detectable treatment effect.
- Enrichment: The recruitment into the trial can be increased within specific population subgroups, for example if an intervention appears to be more effective within the subgroup.^20^

To ensure trial integrity, a cornerstone of adaptive designs is the pre‐specification of what and how adaptations will be made and when scheduled interim analyses will occur. These details should be outlined in the trial protocol and the statistical analysis plan prior to undertaking any analyses. Furthermore, the interim analyses should be conducted and viewed by people independent of the trial, with the trial team remaining blinded to interim results until the end of the trial (or the end of the relevant part of a platform trial, see below).

Adaptive designs offer many advantages over fixed designs, including reduced participant numbers, cost and time, and ethical advantages of fewer participants being exposed to potentially inferior interventions.^21^ The disadvantages are adaptive trials have greater data management and statistical needs throughout the trial, planning the study typically requires computer simulation to estimate the power and expected sample size,^22^ they are more operationally and logistically complex, and the statistical analysis requires careful planning to avoid introducing bias.^23^, ^24^

Adaptive designs are useful when the outcome of interest is relatively short‐term compared to the recruitment period and when it would be advantageous to reach a conclusion as soon as possible. Adaptive designs are less useful when the outcomes take a long time to observe, when dropping arms early may be controversial (e.g. before a safety conclusion can be satisfied), and when increased practical complexity overshadows theoretical efficiency gains.^25^

Case study: The Established Status Epilepticus Treatment Trial (ESETT) was an investigator‐initiated, multicentre, randomised, comparative‐effectiveness trial of levetiracetam, fosphenytoin, and valproate for patients with established status epilepticus in the ED.^26^ The trial involved response adaptive randomisation and allowed for early stopping for superiority or futility based on interim analyses of the primary outcome at 400, 500, 600, and 700 enrolments (max 720 enrolments).^26^

### Platform trials

Platform designs (also referred to as master protocol designs) are an adaptive design that have emerged as a major innovation with global impact over the past two decades, expedited by the COVID‐19 pandemic for which decisions regarding vaccine approval and treatment were required urgently.^27^

A platform trial is an adaptive multi‐arm, multi‐stage trial that allows multiple interventions, across multiple domains (treatment modalities), to be evaluated simultaneously against a common control, in multiple subgroups of participants (referred to as strata). They typically allow early stopping for each intervention (and possibly response adaptive randomisation), have the flexibility to add new interventions and, notionally at least, are designed to operate in perpetuity^28^, ^29^, ^30^ (Fig. 1d).

Platform trials are governed by a core (or ‘master’) protocol supported by several appendices. The master protocol documents the core aims, outcomes, decision rules, estimands, and governance structures. The core protocol is often supported by a statistical appendix that describes the statistical aspects of the study. Both the core protocol and statistical appendix are ideally immutable over time, although may be modified with appropriate approvals. Additional appendices describe ‘non‐core’ aspects of the trial such as intervention‐ or domain‐specific appendices, and other population‐specific or substudy appendices. Many other shared documents support the operation of the trial, including data management plans, guidance documents for interim analyses, simulation reports (updated as the trial makes major adaptations), and domain specific statistical analysis plans that provide specific analytical guidance after conclusion is reached in the domain.

Platform trials have many advantages over conventional designs.^31^ By sharing infrastructure across interventions/domains/strata, platform trials benefit from economies of scale and provide the critical mass of resources needed to pursue important clinical questions. Allowing new treatments to be incorporated into the platform avoids costly duplication of conducting trials either independently or in sequence. Finally, methods can be used to partially pool data across related interventions or strata so that fewer participants are required to reach conclusions; this can be particularly important for underrepresented populations or rare diseases that may go otherwise unstudied.

Despite the advantages, platform trials face challenges.^32^ Operationally, they are complicated and costly to establish, and often require extensive collaboration and coordination across many sites globally. They are highly reliant on specialised statistical expertise throughout the trial, often involving extensive computer simulation to assist with trial design, and development and application of complicated analysis models. There also remain unanswered questions regarding their analysis, for example how to analyse non‐concurrently randomised data^33^, ^34^ and what are the optimal data pooling strategies.^35^

Case study: A well‐known platform trial is the Randomised, Embedded, Multifactorial Adaptive Platform Trial for Community Aquired Pneumona (REMAP‐CAP) trial.^36^ REMAP‐CAP aims to reduce 90‐day all‐cause mortality in participants with severe community‐acquired pneumonia and includes response‐adaptive randomisation and early stopping for superiority, inferiority, or equivalence. At the time of writing, REMAP‐CAP is active in 298 sites, with approximately 24 000 randomisations (across approximately 14 000 patients) with 66 interventions current or completed across 18 domains.^37^

## Conclusion

This paper provides an overview of innovative designs that are being used in modern evidence‐based medicine. We focus on novel cluster randomised, sequential and platform designs given their potential in emergency medicine, although acknowledge there are other designs we have not covered, for example micro‐randomised trials,^38^ and N‐of‐1 studies^39^ which are more common in chronic conditions. The use of innovative designs is increasing but there is still a need for greater awareness of these designs and when they may and may not be useful to ensure the best design is being used to address the research question of interest. The main drawback of the designs presented here are that they are complex to design and analyse, and are highly reliant on specialist expertise, in particular in statistics. It is imperative that we develop capacity in these innovative designs to enable researchers to use the most appropriate design to address their research question.

The world of evidence‐based medicine is changing and it is important that researchers are aware of the advances in trial design so that they can design their research studies to answer important research questions as effectively and efficiently as possible. We hope that this overview goes some way to achieving this in emergency medicine.

### Competing interests

None declared.

## Data Availability

Data sharing not applicable to this article as no datasets were generated or analysed during the current study.
